# Which Personal and Organizational Factors Influence the Organizational Commitment and Job Satisfaction of Shipyard Blue-Collar Workers?

**DOI:** 10.3390/ijerph19084849

**Published:** 2022-04-16

**Authors:** Adela Reig-Botella, Miguel Clemente, Sarah Detaille, Annet H. de Lange, Jaime López-Golpe

**Affiliations:** 1 Department of Human Resource Management, HAN University of Applied Sciences in Nijmegen, 6525 EJ Nijmegen, The Netherlands; sarah.detaille@han.nl (S.D.); annet.delange@ou.nl (A.H.d.L.); 2 Department of Psychology, Campus Industrial de Ferrol, Universidade da Coruña, 15403 Ferrol, Spain; 3 Department of Psychology, Campus de A Coruña, Universidade da Coruña, 15701 A Coruña, Spain; miguel.clemente@udc.es (M.C.); jaime.lopez.golpe@gmail.com (J.L.-G.); 4 Faculty of Psychology, Open University Heerlen, 6419 AT Heerlen, The Netherlands; 5 Norwegian School of Hotel Management, University of Stavanger, 4021 Stavanger, Norway; 6 Faculty of Psychology, Norwegian University of Science and Technology, 7491 Trondheim, Norway

**Keywords:** blue-collar workers, job satisfaction, job commitment, personal and organizational factors, shipyard

## Abstract

Background: The purpose of this research was to analyze which personal and organizational factors are related to organizational commitment and job satisfaction of shipyard workers who work in different auxiliary shipyard military companies in the north of Spain. Methods: In total, 567 shipyard workers participated in this cross-sectional survey study. The ages were between 19 and 64 (M = 39.36, SD = 10.01), males 82.52%, females 17.48%. We used a survey that included questions about personal and organizational factors such as physical environment, occupational risks, and psychosocial risks, in addition to the job commitment and job satisfaction scales. Results: The results of this study show that job commitment is significantly related to a higher age, lower education, and environmental risk (low vs. high). Furthermore, job satisfaction (high vs. low) and organizational commitment (high vs. low) is related with environmental risk (low vs. high). Job commitment is also higher in workers with a low educational level and older workers. Job satisfaction is lower in workers with a high educational level. Conclusions: This study shows that different personal and environmental factors influence the shipyard workers’ organizational commitment and job satisfaction.

## 1. Introduction

Exposure to environmental risk, especially psychosocial risk in the work environment, is one of the biggest challenges to the occupational safety and health of shipyard workers [1]. Shipyard employees endure particularly hard working conditions which are characterized by, among other factors, demanding physical activities and extreme (high or low) temperature [2]. Several studies show that levels of job commitment and job satisfaction may deteriorate if shipyard workers have been in the same shipyard company for a long duration and their working conditions have been disregarded [2,3]. This study focused on further analyzing which personal and organizational factors are related to job commitment and job satisfaction among shipyard workers within the auxiliary shipyard military industry in northern Spain to determine ways of intervening and preventing drop-outs.

### 1.1. Theoretical Background

No study to date has examined the role of specific personal and organizational factors in sustaining the commitment and well-being of Spanish shipyard workers. To further investigate the influence of these variables among shipyard blue-collar workers, we therefore conducted a new cross-sectional survey study. Before addressing the specific research questions and hypotheses of the current study, the relevant theories and concepts of job satisfaction, organizational commitment, and psychosocial risk will be developed.

Previous research has indicated that content and enthusiastic staff are essential for staff retention and continuity at the workplace [3] and may also reduce occupational accidents within shipyard work [4]. Psychosocial factors refer, in this context, to working conditions, work environment, characteristics and functions of employee tasks, interpersonal relationships at work, the environment outside the workplace, and employee personality. Some psychosocial factors such as harsh working conditions or lack of social support at work can negatively affect employee physical and mental health and can be considered an occupational risk factor [5,6]. The perception of occupational health risks will significantly and negatively affect employees’ job satisfaction [7].

Earlier research has shown that job satisfaction is related to the environmental risk perception, especially in the case of negative working conditions such as being exposed to loud noises, vibrations, and cold temperatures [8]. Furthermore, studies have also found effects of personal factors such as age education and time perspective [9] in influencing the well-being and satisfaction of shipyard worker.

*Personal and organizational factors and job satisfaction*. Job satisfaction is a perception based on experiences linked to one’s work and the states of mind they cause [10], and is often used as an intuitive meter of career success [11]. A favorable correlation between career suitability and job satisfaction can assist in retaining older employees [12]. Certain job features associated with the age of a worker or the time with a company are determinant in predicting signs of a lack of occupational well-being, and these can affect worker engagement, job satisfaction, and mental fatigue [13].

Other factors can also play a role in the job satisfaction of older and low-skilled employees. Research by Gonzalez et.al. 2016 found that job-security can play a role in job-satisfaction as older and low-skilled employees are more likely to express higher levels of gratitude for the job since they have been able to secure and maintain it [14].

Organizational commitment illustrates the level to which workers identify with the organization for whom they work, how involved in the organization they are, and whether they intend to leave the institution [15]. As stated by Hwang and Cha [16], the notion of organizational commitment enhances the natural enthusiasm of workers. Individuals with high organizational commitment have a tendency towards strong levels of loyalty towards the institution and have high productivity [17].

This concept of organizational commitment is thought to be a supplement to job satisfaction, because it relates to the positive attitude a worker has, not of one’s own role, but towards the institution itself, and it is an underlying feature of somebody’s behavior in an organization.

Staff who are more devoted to a company are less prone to quit their job as they have an emotional connection to their company, indicated by the psychological bond the worker has to the company and their readiness to make sacrifices for it [3]; the organizational support perceived by employees and their organizational commitment are important factors [18]. Some studies further the hypothesis that job satisfaction anticipates organizational commitment [19]. This is the psychological link between staff and their organization: the greater the satisfaction, the greater the motivating force. Moreover, the happiest workers with intellectual disabilities are the ones who are the most the motivated [20,21] and committed to the organization.

The model of organizational commitment (TCM) has three elements with corresponding scales to measure each of them. Affective commitment refers to the feeling towards the institution that involves a staff member’s positive mental bond with their organization [22]. It is considered to be the attitude towards the organization [23]. Workers see the organization’s aims as complementary to their own, and are more willing to invest their own time and effort into it [24]. In contrast, continuance commitment happens when employees think they have to continue to be part of an organization (e.g., they need to work for a salary); therefore, there is an exchange of involvement for rewards, with high costs involved in leaving [25]. Finally, normative commitment refers to employees’ feelings of obligation to remain in the organization. Both continuance and normative commitment focus more on employees’ attitudes toward specific behaviors, such as turnover behavior [17]. The result of a dearth of organizational commitment includes absence for illness, high turnover, reduced job performance, a reduced organizational citizenship behavior, higher stress levels, and work–life friction [23].

Dhaenens et al. [26] stated that organizational commitment may be thought of as the central feature of human resource management since committed workers are more content, higher performing, stronger organizational citizens, and leave the organization less frequently [27].

Institutions that cultivate organizational commitment significantly reduce elective turnover and minimize human resource costs [28]. A major method of cultivating staff is by mentoring. This can also be affected by environmental factors.

Supplementary factors related to organizational commitment are reinforced by other research. An element that differentiates between the public and the private sector is whether the employee feels their work is worthwhile and motivates workers [29,30].

Nevertheless, a major impact of various aspects of the working environment was discovered in relation to job satisfaction [31]. Employees trained in social sciences gave more favorable marks to the characteristics of their jobs than employees with technical skills. The more highly educated (e.g., university educated) the workers, the less absorbed they are in their work and the less they are concerned about the characteristics of their job.

*Aging, work outcomes among shipyard workers.* Earlier research has shown that there is a major correlation between job satisfaction and age, whereby the older the worker, the less content they are with their job and the less concerned they are about the different elements of their job [3]. Therefore, we also further examined relations between aging and commitment among shipyard workers.

The workforce in Western countries is becoming older and employees are retiring at different ages, a process that is expected to persist due to the higher life expectancy [32] and increasing retirement age [33]. This shift has caused demographic, sociocultural, and economic issues, especially for blue-collar workers who perform predominantly manual labor and have seen their retirement age increased [34]. Furthermore, the economic crisis has resulted in workers delaying their retirement due to lack of financial resources to sustain it [35].

In Spain, the government policy regarding retirement makes the following classification: (1) Ordinary retirement: this occurs when one can apply for a retirement pension after reaching the legal retirement age. The legal retirement age is set at 65 years old, as long as one can prove to have contributed to the fiscal system for 37 years. This system implies that, in order to claim a retirement pension, the claimant’s age and the contributions paid over his/her working life will need to be taken into account. (2) Flexible retirement: in order to access this type of retirement, one can combine receiving a part of the pension with part-time work (reducing the full working day between 25% and 50%). The pension is reduced proportionally, and the condition of “pensioner” is not altered. (3) Partial retirement: in the case where one has not reached the legal retirement age, there is the possibility to combine a part-time employment contract with receiving part of the retirement pension, reducing the working day by 25% to 50% and meeting the rest of the conditions required for ordinary retirement [36].

Another factor to take into account is older workers, who are classified as those aged from their 40s to over retirement age, who are increasingly prompted to stay in work longer [37]. These workers’ collective expertise and experience are highly valued by many institutions, who are therefore interested in keeping them on [12], as they are more satisfied compared to young workers [38].

Successful aging at work depends on different factors such as mental and physical health, stable relationships, professional development, personal security, and accomplishment of personal objectives. These are shared duties between staff and management [39] as is the case with stability or improvement of outcomes, for example, motivation, achievement, welfare [40], an individual’s continuous successful adjustment to age-related alterations, and demands of the working environment [41] throughout the working life. Moreover, flourishing at work involves a higher-than-expected level of productivity in the workplace even when measures of attitude, e.g., organizational commitment and job satisfaction, are excluded [42].

There is also a gap between notions of aspiration and prospects of achievement among younger and older staff, with younger staff being more content with just having succeeded in getting a job rather than being satisfied with the work they are doing. As working conditions within the shipyard are harsh [43], once they spend longer in the organization, they become less satisfied with their job and their contentment with aspects of their work is reduced.

### 1.2. Objectives and Hypothesis

The research goal of this study was to analyze if certain personal/demographic and environmental risk factors are related to a higher organizational commitment and job satisfaction in shipyard workers in order to identify possible vulnerable target groups within the shipyard who are at risk of being demotivated.

We formulated the following hypotheses in the current research:

**Hypothesis** **1.** *Affective commitment is related to age (high vs. low) and educational level (low vs. high) but is not related to gender.*

**Hypothesis** **2.** *Organizational commitment (high vs. low) is significantly positively related to a low environmental risk perception.*

**Hypothesis** **3.** *Normative Commitment is positively related to age, educational level, and risk.*

**Hypothesis** **4.** *Job satisfaction (high vs. low) is significantly positively related to a low environmental risk perception.*

**Figure 1:**
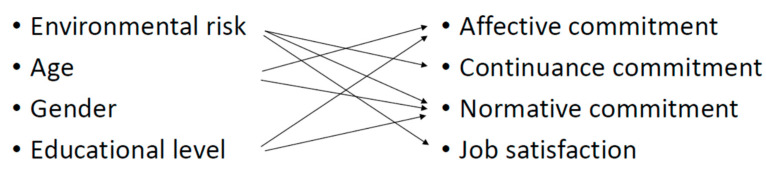
Hypotheses about the predictor variables.

A summary of the relationships found between the hypothesized model predictor and dependent variables can be seen below (Figure 1).

## 2. Materials and Methods

### 2.1. Participants

The total population consisted of 1538 people who worked for 33 companies of the auxiliary industry contributing services in a shipyard military industry. Of this population, a meaningful sample of 567 participants—472 male (82.52%); 95 female (17.48%)—who are employees of a shipyard in northern Spain responded to our cross-sectional survey study, with a mean age of 38 years old, seniority in the organization 8.5 years, and 14 years work experience. The respondents (incidental sample) voluntarily participated. They were given the survey and informed consent form in person that explained the purpose of the study, assured them of their anonymity, and provided information about how to contact researchers to answer any questions or exercise their rights to delete the information provided. They were requested to return the responses in two weeks’ time. The questionnaire was administered only once. Data collection took place between October and December 2021. This research was approved by the Ethic Committee of the Research Group on Criminology Legal Psychology and Criminal Justice of the University of the corresponding author on 7 February 2022 (56/22) with the title “Occupational Health Psychology and naval sector”.

After age and gender, education level was also analyzed: 182 respondents (32%) studied and completed primary education, 240 respondents (42%) studied and completed secondary education, and 129 respondents (22%) attended university. Regarding the employment situation and the type of contracts, 136 (24%) have a permanent contract, 114 (20%) a temporary contract, and 132 (23%) a work contract.

### 2.2. Data Collection

An ad hoc questionnaire, prepared specifically for this research, was designed, which included a questionnaire of socio-demographic variables in addition to questions about affective, continuance, and normative commitment, job satisfaction, physical environment, occupational risks, and psychosocial risks. The questionnaire was comprised of 50 questions and separated into six sections.

Socio-demographic variables: age, gender, marital status, level of studies, type of work contract, years of work experience, salary, current role, activity of the organization. With the exception of the socio-demographic variables, we used the following instruments to create the different sections of the questionnaire.

▪ Section I: “Affective Commitment Scale” (6 questions, items 1 to 6).
▪ Section II: “Continuance Commitment Scale” (8 questions, items 7 to 14).

We used the Organizational Commitment Questionnaire “Meyer, Allen and Smith” (1993) [44].

▪ Section III: “Normative Commitment Scale” (6 questions, items 15 to 20).

Taking the Organizational Commitment Questionnaire “Meyer, Allen and Smith” (1993) as a starting point, we created the 6 items of the Section III of the ad hoc questionnaire.

▪ Section IV: “Job Satisfaction” (10 questions, items 21 to 30).

Taking The S20/23 Job Satisfaction Questionnaire. Meliá, J.L. and Peiró, J.M. (1989) [45] as a basis, we created the 10 items of the ad hoc questionnaire.

▪ Section V: Physical Environment (8 questions, items 31 to 38).

Taking the self-assessment of working conditions. INSHT-NTP 182 (1986) [46] as a starting point, we created the 8 items of the ad hoc questionnaire.

▪ Section VI: Occupational Risks (6 questions, items 39 to 44) and Psychosocial Risks (6 questions, items 45 to 50).

Taking the CoPsoQ-Istas 21 (2010) [47] as a basis, we created the 12 items of the ad hoc questionnaire.

A Likert scale with five alternatives was used. A value of 1 indicated completely disagree, 2—disagree, 3—undecided, 4—agree, and a value of 5 indicated completely agree. The reliability of each of the variables (and corresponding scales) that made up the questionnaire was calculated using the Cronbach’s Alpha index.

The results obtained (Table 1, Table 2) were adequate. The reliability of all the scales and subscales of the measuring instrument was adequate.

The data was entered into an Excel spreadsheet, then converted into the IBM SPSS Program, v.26. To analyze the data, three multiple linear regressions were performed. In the first, the dependent variable was affective commitment; in the second—continuance commitment; in the third—normative commitment; and in the fourth—job satisfaction.

In all of these, the predictive or independent variables were age, gender, educational level, and level of occupational risk. The coding of the ordinal variables in order to carry out the regression technique was: gender (Male = 0, Female = 1) and educational level (1 = studied and completed primary education, 2 = studied and completed secondary education, and 3 = attended university). All significances were applied for *p* < 0.05

## 3. Results

Table 2 specifies the Pearson correlation coefficients found in the study variables.

**Table 2:** Pearson’s Correlations between study variables.

Variable	Continuance Commitment	Normative Commitment	Job Satisfaction	Environmental Risk	Age	Gender
Normative commitment	0.427 **					
Job satisfaction	0.393 **	0.616 **				
Environmental risk	−0.160 **	−0.293 **	−0.542 **			
Age	0.076	0.129 **	0.094 *	−0.082		
Gender	0.032	−0.014	0.036	−0.066	−0.127 **	
Education level	−0.085 *	−0.103 *	0.026	−0.270 **	−0.178 **	0.366 **

Note: * Correlation is significant at the 0.05 level (2-tailed). ** Correlation is significant at the 0.01 level (2-tailed).

### 3.1. Organizational Commitment

The multiple linear regression carried out with the dependent variable Affective Commitment did not obtain significant results (none of the sociodemographic variables was significantly explanatory of the same). Due to this fact, only one of the tables obtained in this regard is presented (Table 3).

The results obtained for the dependent variable Organizational Commitment (continuance commitment) is presented first. The first of the tables (see Table 4) shows two possible predictive models; the first makes the variables education level, age, and gender constant, and the second adds environmental hazards to the model. Both models significantly predict continuance commitment, although the prediction of environmental hazard is higher.

To ensure this significance, the ANOVA test (Table 5) was applied to verify the prediction effectiveness of the models, and the extent to which the prediction was more powerful if environmental hazard was added. Table 6 adds specific information on which variables are more explanatory of the determination of the continuance commitment. This table establishes that, for model 1, which excludes environmental hazard, the only significant predictor variable is educational level (negative), and in model 2, which includes environmental hazard, the two predictor variables are educational level and environmental hazard, both in a negative sense (see Table 6).

### 3.2. Normative Commitment

With regard to normative commitment, both Table 7 (predictive model obtained through linear regression) and Table 8 (verification of the model through Analysis of Variance) show how age becomes a key predictor, whereas this is not the case for gender (which was significant for the continuance commitment). Furthermore, as in the case of the previous dependent variable, when the risk variable is introduced into the model, it also becomes a predictor. By comparison, if we examine the weight of each variable in the prediction (Table 9), three significant predictor variables can be observed: age, educational level, and risk. Age obtains a positive score (the older the worker, the higher the degree of prediction of normative commitment), whereas the educational level and the risk level obtain a negative score (the lower the educational level and the lower the risk level, the higher the normative commitment).

### 3.3. Job Satisfaction

Finally, the variables that predict the greater or lesser presence of job satisfaction in workers were analyzed. One of the models resulting from the linear regression is significant (see Table 10, model b), namely, the model in which the variables education level, age, and gender (a) remain constant. The second model is significant, adding the environmental hazard variable as a constant. Therefore, the environmental hazard variable becomes fundamental in the prediction.

Subsequently, an ANOVA test was performed (Table 11), which verified the prediction. The weight of each significant variable in the prediction can be seen in Table 12. This table verifies age is predictive in the first model but not in the second, where the educational level and the risk level are predictors. In general, workers with a lower educational level, in addition to those who perceive a lower level of risk, tend to show greater satisfaction.

It should be noted that there is a direct predictive negative relationship between the variables analyzed (continuance commitment, normative commitment, and job satisfaction) and perceived environmental hazards.

## 4. Discussion

This study shows that personal variables such as age, education, and perception of risk at work significantly influence the shipyard workers’ organizational commitment and job satisfaction. Few studies to date have examined these relations among shipyard workers [5,10,48]. The following hypotheses were formulated and tested in this study.

**Hypothesis** **5.** *Affective commitment will vary according to age in a positive way, and Education level and Environmental risk in a negative way. This hypothesis was not fulfilled, since none of the predictor variables analyzed was significant*.

**Hypothesis** **6.** *Organizational commitment is significantly positively related to a low environmental risk perception. This hypothesis was fulfilled in one of its predictors, since the results obtained indicate that educational level and environmental hazard were both relevant in a negative sense. Workers with a lower educational level, in addition to those who perceive a lower level of risk, tend to show greater satisfaction*.

**Hypothesis** **7.** *Normative commitment will be positively related to age, educational level, and risk. This hypothesis was fully supported. Age obtained a positive score (the older the worker, the higher the degree of prediction of normative commitment), whereas the educational level and the risk level obtained a negative score (the lower the educational level and the lower the risk level, the higher the normative commitment)*.

**Hypothesis** **8.** *Job satisfaction is significantly positively related to a low environmental risk perception. This hypothesis was partially fulfilled, since the data show that workers with a lower educational level, in addition to those who perceive a lower level of risk, tend to show greater satisfaction. This study shows that the perception of lower levels of environmental risk increases organizational commitment and job satisfaction in older and less educated shipyard employees*.

**Figure 2:**
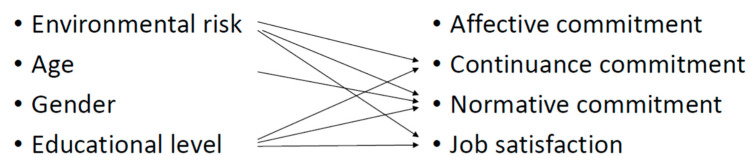
Relationships found between the predictor and dependent variables.

A summary of the relationships found between the predictor and dependent variables can be seen below (Figure 2)

The findings of this study correspond with previous research. Other studies found that affective commitment correlates more strongly with absence, performance, and organizational citizenship behaviours [49]. In the case of the other variables, results are found to be in accordance with the literature on the matter which revealed, generally speaking, that occupational commitment is related to personal antecedents such as type of occupation. Occupational commitment is stronger for blue-collar and non-professional white-collar workers than for professional workers [50]. A reason for this may be that, within the shipyard, blue-collar workers are grateful that they have a sustainable and fixed job. Other studies have found that even though low-skilled workers experience a lack of job-satisfaction due to the harsh working conditions, they can still perceive a high organizational commitment because they are grateful for being employed, especially in geographic areas where there is a high level of unemployment [14]. Another explanation for the high organizational commitment in older shipyard workers is the fact that family members also work at the shipyard, and that generation after generation work at the same shipyard. The shipyard organization has become part of the family culture of the worker.

Moreover, blue-collar workers experience less job satisfaction than their white-collar colleagues, such as in terms of pay and the meaning of work itself [51]. The reason for the lack of job satisfaction may be the harsh working conditions at the shipyard. Shipyard staff are exposed to noise and air pollution [2]. Moreover, the type of leadership and social influence at the workplace also impacts organizational commitment and job satisfaction at the workplace. Several theorists have conjectured about the cause-and-effect nature of the relationship between shared vision and affective commitment and job satisfaction [52]. A positive relationship has been found [53] between a leader-provided shared vision and the job satisfaction of workers in the service industry. Organizations should pay attention to the personal and organizational factors that influence the relationships with employees since committed employees influence the performance and productivity of organizations [54].

This study has several limitations which hinder the generalization of results. A limitation of the study is that we are unable to verify if variables other than age, level of education, and perception of environmental risk influence the organizational commitment and job satisfaction of workers. Other aspects such as type of job, social support in the workplace, time perspective [43], and lack of autonomy may also influence the level of organizational commitment in highly educated and younger workers. Rather than the level of education, the main problem is probably the type of job and related working factors, such as working conditions and employment conditions. In the future, more research is needed to test the relationship of these factors, for instance by using the Job–Demand–Resources model. Moreover, recent research has shown that workers who have been working for more than 10 years at the shipyard have a more fatalistic and negative time perspective than workers who have been working fewer years at the shipyard. This factor may also explain the difference in job satisfaction between older and younger workers at the shipyard. Furthermore, the psychological contract of older and younger workers may be different at the shipyard. Differences in psychological contract can also be analyzed in future work. Additional qualitative interviews may also help to explain the mechanisms related to job satisfaction and environmental safety at shipyards.

Additionally, for future research, it is necessary to verify the validity of the instruments that were used in this study. These were verified regarding their reliability and include scientifically verified instrument items, but were not tested in this study for their validity.

Nonetheless, the study gives insight into the possible factors that may influence organizational commitment and job satisfaction of shipyard workers. We suggest that it is important to train supervisors and occupational health professionals to recognize symptoms of negative organizational commitment and lack of job satisfaction in both younger and highly educated workers at shipyards. In general, organizations need to observe to the effects of a negative time perspective of shipyard workers as this can result in mental problems such as burnout [43].

HRM practices within the shipyard should pay attention to different socio-demographic factors and the different job demand interactions within the shipyard. The HR instruments within the shipyard should be targeted to different groups of workers [55]. Furthermore, there is a necessity to intervene within the shipyard on the basis of participation so that blue-collar workers feel more involved in the process of actions taken at the workplace, to improve the organizational commitment and work satisfaction of these workers, and to provide measures to promote a safe and engaged workplace.

## 5. Conclusions

This study shows that different personal and environmental factors, growth opportunities, managerial support [56], and psychological well-being [57] influence the motivation and job satisfaction [58] of workers at the shipyard. The mechanisms are different for low-skilled workers or higher educated workers. The level of education is related to working factors characterized by physically highly demanding work, low autonomy, and lack of social support. Such working conditions probably influence job satisfaction and environmental risk perception in a negative way. The continuance commitment at work is higher if shipyard blue-collar workers have lower educational levels and perceptions of environmental hazards are lower. The normative commitment is greater in older workers, those with a lower educational level, and when the perceptions of environmental hazards and level of risk are lower. The subjective psychological contract may be different for older or younger workers, and for workers with a different type of educational level [59]. Workers with a lower educational level, and those who perceive a lower level of environmental risk at work, tend to show a greater job satisfaction. The perception of lower levels of environmental hazards increases the continuance commitment, the normative commitment, and the level of job satisfaction.

## Figures and Tables

**Table 1:** Cronbach’s Alpha Index (reliability).

Affective Commitment	0.798
Continuance commitment	0.884
Normative commitment	0.871
Job satisfaction	0.868
Physical environment	0.926
Risk scale (Global)	0.879
Occupational risks subscale	0.802
Psychosocial risks subscale	0.922

**Table 3:** Predictive model of affective commitment.

Model	R	R^2^	Adjusted R^2^	Std. Error of the Estimate	Change Statistics				
					R^2^ Change	F Change	df1	df2	Sig. F Change
1	0.147 ^a^	0.022	0.016	0.34455	0.019	2.652	3	498	0.123
2	0.256 ^b^	0.065	0.058	0.23421	0.022	16.259	1	497	0.232

Note: ^a^ Predictors: (Constant), Education level, Age, Gender; ^b^ Predictors: (Constant), Education level, Age, Gender, Environmental risk; Std.: standard; df: degrees of freedom; Sig.: Signification.

**Table 4:** Predictive model of continuance commitment.

Model	R	R^2^	Adjusted R^2^	Std. Error of the Estimate	Change Statistics				
					R^2^ Change	F Change	df1	df2	Sig. F Change
1	0.147 ^a^	0.022	0.016	0.68286	0.022	3.657	3	498	0.012
2	0.256 ^b^	0.065	0.058	0.66809	0.044	23.260	1	497	0.000

Note: ^a^ Predictors: (Constant), Education level, Age, Gender; ^b^ Predictors: (Constant), Education level, Age, Gender, Environmental risk; Std.: standard; df: degrees of freedom; Sig.: Signification.

**Table 5:** ANOVA model for continuance commitment.

Model		Sum of Squares	df	Mean Square	F	Sig.
a	Regression	5.115	3	1.705	3.657	0.012 ^a^
	Residual	232.218	498	0.466		
	Total	237.333	501			
b	Regression	15.497	4	3.874	8.680	0.000 ^b^
	Residual	221.836	497	0.446		
	Total	237.333	501			

Note: ^a^ Predictors: (Constant), Education level, Age, Gender; ^b^ Predictors: (Constant), Education level, Age, Gender, Environmental risk; df: degrees of freedom; Sig.: Signification.

**Table 6:** Coefficients and significance for the dependent variable continuance commitment.

Model		Unstandardized Coefficients		Standardized Coefficients	t	Sig.
		B	Std. Error	Beta		
a	(Constant)	2.805	0.179		15.635	0.000
	Age	0.005	0.003	0.069	1.526	0.128
	Gender	0.154	0.089	0.084	1.737	0.083
	Education level	−0.119	0.045	−0.129	−2.652	0.008
b	(Constant)	3.467	0.223		15.560	0.000
	Age	0.003	0.003	0.044	0.994	0.321
	Gender	0.165	0.087	0.089	1.894	0.059
	Education level	−0.179	0.046	−0.193	−3.913	0.000
	Environmental risk	−0.167	0.035	−0.218	−4.823	0.000

Note: a Predictors: (Constant), Education level, Age, Gender; b Predictors: (Constant), Education level, Age, Gender, Environmental risk Std.: standard; Sig.: Signification.

**Table 7:** Predictive model of normative commitment.

Model	R	R^2^	Adjusted R^2^	Std. Error of the Estimate	Change Statistics				
					R^2^ Change	F Change	df1	df2	Sig. F Change
a	0.165 ^a^	0.027	0.021	0.79913	0.027	4.444	3	479	0.004
b	0.376 ^b^	0.141	0.134	0.75163	0.114	63.456	1	478	0.000

Note: ^a^ Predictors: (Constant), Education level, Age, Gender; ^b^ Predictors: (Constant), Education level, Age, Gender, Environmental risk; Std.: standard; df: degrees of freedom; Sig.: Signification.

**Table 8:** ANOVA model for normative commitment.

Model		Sum of Squares	df	Mean Square	F	Sig.
a	Regression	8.513	3	2.838	4.444	0.004 ^a^
	Residual	305.894	479	0.639		
	Total	314.407	482			
b	Regression	44.362	4	11.091	19.631	0.000 ^b^
	Residual	270.045	478	0.565		
	Total	314.407	482			

Note: ^a^ Predictors: (Constant), Education level, Age, Gender; ^b^ Predictors: (Constant), Education level, Age, Gender, Environmental risk; df: degrees of freedom; Sig.: Signification.

**Table 9:** Coefficients and significance for dependent variable normative commitment.

Model		Unstandardized Coefficients		Standardized Coefficients	t	Sig.
		B	Std. Error	Beta		
a	(Constant)	2.139	0.211		10.145	0.000
	Age	0.010	0.004	0.129	2.812	0.005
	Gender	0.086	0.104	0.040	0.823	0.411
	Education level	−0.100	0.053	−0.092	−1.870	0.062
b	(Constant)	3.397	0.253		13.400	0.000
	Age	0.008	0.004	0.093	2.147	0.032
	Gender	0.102	0.098	0.048	1.044	0.297
	Education level	−0.219	0.052	−0.202	−4.178	0.000
	Environmental risk	−0.316	0.040	−0.354	−7.966	0.000

Note: a Predictors: (Constant), Education level, Age, Gender; b Predictors: (Constant), Education level, Age, Gender, Environmental risk; Std.: standard; Sig.: Signification.

**Table 10:** Predictive model for job satisfaction.

Model	R	R^2^	Adjusted R^2^	Std. Error of the Estimate	Change Statistics				
					R^2^ Change	F Change	df1	df2	Sig. F Change
a	0.111 ^a^	0.012	0.006	0.77507	0.012	1.889	3	454	0.131
b	0.569 ^b^	0.324	0.318	0.64200	0.312	208.709	1	453	0.000

Note: ^a^ Predictors: (Constant), Education level, Age, Gender; ^b^ Predictors: (Constant), Education level, Age, Gender, Environmental risk; Std.: standard; df: degrees of freedom; Sig.: Signification.

**Table 11:** ANOVA model for job satisfaction.

Model		Sum of Squares	df	Mean Square	F	Sig.
a	Regression	3.405	3	1.135	1.889	0.131 ^a^
	Residual	272.730	454	0.601		
	Total	276.135	457			
b	Regression	89.426	4	22.357	54.242	0.000 ^b^
	Residual	186.709	453	0.412		
	Total	276.135	457			

Note: ^a^ Predictors: (Constant), Education level, Age, Gender; ^b^ Predictors: (Constant), Education level, Age, Gender, Environmental risk; df: degrees of freedom; Sig.: Signification.

**Table 12:** Coefficients and significance for dependent variable job satisfaction.

Model		Unstandardized Coefficients		Standardized Coefficients	t	Sig.
		B	Std. Error	Beta		
a	(Constant)	2.679	0.216		12.414	0.000
	Age	0.008	0.004	0.106	2.217	0.027
	Gender	0.096	0.103	0.047	0.933	0.351
	Education level	0.027	0.053	0.025	0.498	0.619
b	(Constant)	4.623	0.224		20.662	0.000
	Age	0.004	0.003	0.049	1.236	0.217
	Gender	0.152	0.086	0.074	1.776	0.076
	Education level	−0.160	0.046	−0.152	−3.465	0.001
	Environmental risk	−0.494	0.034	−0.583	−14.447	0.000

Note: a Predictors: (Constant), Education level, Age, Gender; b Predictors: (Constant), Education level, Age, Gender, Environmental risk; Std.: standard; Sig.: Signification.

## Data Availability

The data presented in this study are available on request from the corresponding author.
